# Characteristics of wild polio virus outbreak investigation and response in Ethiopia in 2013–2014: implications for prevention of outbreaks due to importations

**DOI:** 10.1186/s12879-017-2904-9

**Published:** 2018-01-05

**Authors:** Ayesheshem Ademe Tegegne, Fiona Braka, Meseret Eshetu Shebeshi, Aron Kassahun Aregay, Berhane Beyene, Amare Mengistu Mersha, Mohammed Ademe, Abdulahi Muhyadin, Dadi Jima, Abyot Bekele Wyessa

**Affiliations:** 1 World Health Organization Country Office, P. O. Box 3069, Addis Abba, Ethiopia; 2 Inter-Country Support Team eastern and Southern Africa, IST, Harare, Zimbabwe; 3 Ethiopian Public Health Institute, Addis Ababa, Ethiopia; 4 World Health Organization Technical Support Team, Jijiga, Somali Region Ethiopia

**Keywords:** Wild polio virus outbreak, Importation, Outbreak, Horn of Africa, Pastoralist, Immunization, Surveillance

## Abstract

**Background:**

Ethiopia joined the Global Polio Eradication Initiative (GPEI) in 1996, and by the end of December 2001 circulation of indigenous Wild Polio Virus (WPV) had been interrupted. Nonetheless, the country experienced multiple importations during 2004–2008, and in 2013. We characterize the 2013 outbreak investigations and response activities, and document lessons learned.

**Method:**

The data were pulled from different field investigation reports and from the national surveillance database for Acute Flaccid Paralysis (AFP).

**Results:**

In 2013, a WPV1 outbreak was confirmed following importation in Dollo zone of the Somali region, which affected three Woredas (Warder, Geladi and Bokh). Between July 10, 2013, and January 5, 2014, there were 10 children paralyzed due to WPV1 infection. The majorities (7 of 10) were male and below 5 years of age, and 7 of 10 cases was not vaccinated, and 72% (92/129) of < 5 years of old children living in close proximity with WPV cases had zero doses of oral polio vaccine (OPV). The travel history of the cases showed that seven of the 10 cases had contact with someone who had traveled or had a travel history prior to the onset of paralysis. Underserved and inaccessibility of routine immunization service, suboptimal surveillance sensitivity, poor quality and inadequate supplemental immunization were the most crucial gaps identified during the outbreak investigations.

**Conclusion:**

Prior to the 2013 outbreak, Ethiopia experienced multiple imported polio outbreaks following the interruption of indigenous WPV in December 2001. The 2013 outbreak erupted due to massive population movement and was fueled by low population immunity as a result of low routine immunization and supplemental Immunization coverage and quality. In order to avert future outbreaks, it is critical that surveillance sensitivity be improved by establishing community-based surveillance systems and by assigning surveillance focal points at all level particularly in border areas. In addition, it is vital to set up in hard to reach areas a functional immunization service delivery system using the “Reaching Every Child” approach, including periodic routine immunization intensification and supplemental immunization activities.

## Background

The Global Polio Eradication (GPEI) was set up in 1988, when the World Health Assembly (WHA) passed a resolution to eradicate polio by the year 2000 [1]. It is the largest public health initiative ever in the history of global public health. The GPEI assumed the implementation of four basic strategies to achieve global polio eradication: 1) high routine immunization of every child below 1-year-of age with at least three doses of Oral Polio Vaccine (OPV); 2) National Immunization Days (NIDs) targeting children under 5 years old; 3) sensitive surveillance systems and; 4) mop up campaigns in areas where population immunity is low due to sub-optimal routine immunization coverage and presence of cases that are compatible with polio. Due to several challenges, the goal set for 2000 was not met and in 2012, the GPEI developed the Polio Eradication and Endgame Strategic Plan covering the period 2013 to 2018, aimed at a polio-free world by 2018. The End Game Strategy has four main objectives, which are: 1) detection of WPV type 1 due to importation and interruption of transmission, 2) strengthening immunization service and OPV withdrawal, 3) containment of WPV type 2 and certification of polio, and 4) polio legacy planning [2].

In the pre-polio eradication era, polio was generally endemic in many African countries with 1597 polio cases recorded in the continent in 1995 [3]. By the end of 2015, the number of cases in Africa had dropped to zero but in August 2016 Nigeria reported four WPV cases after almost 2 years of polio-free status.

Ethiopia joined the GPEI in 1996 and managed to interrupt transmission of indigenous WPV transmission in December 2001. However, prior to the most recent outbreak in 2013, Ethiopia had five WPV importations between 2004 and 2008. The importation in 2004, which affected the northern part of the country, was genetically linked to the virus that originated from Nigeria through Chad and Sudan, the one in 2005 was genetically linked to the virus circulating in Sudan; and the 2006 importation into the southern and eastern part of Ethiopia was genetically linked with virus circulating in Somalia. The importation in Gambella region in 2008, after 17 months of no WPV circulation, was genetically linked to the virus circulating in southern Sudan [3–6].

In 2013, Horn of Africa(HOA) countries experienced more outbreak associated cases of WPV type 1 than had ever been documented before, and affected Somalia, Kenya, and Ethiopia [7–9]. The first case was detected in Mogadishu in Somalia with date of onset of paralysis on 18th April, 2013; subsequently the outbreak spread to Kenya, where the first confirmed case had onset of paralysis on 30th April 2013. On August 14th, 2013, the Kenya Medical Research Institute (KEMRI) issued an advance notification of a WPV1 case living in Ethiopia, but detected and reported through cross-border notification by the Somalia surveillance team. The WPV1 case had date of onset of paralysis of July 10th, 2013 and was notified 3 months after the detection of a WPV1 outbreak in Somalia. A total of 223 WPV type 1 cases were reported in the Horn of Africa outbreak and the poliovirus was closely linked to the poliovirus circulating in Nigeria [10–14].

We describe the 2013 polio outbreak in Ethiopia with emphasis on the outbreak investigation, risk factors and response efforts made, including a review of the implications of prevention, preparedness, and response to any future importation in sustaining the polio-free status of the country.

## Methods

### Study area and setting

**Fig. 1:**
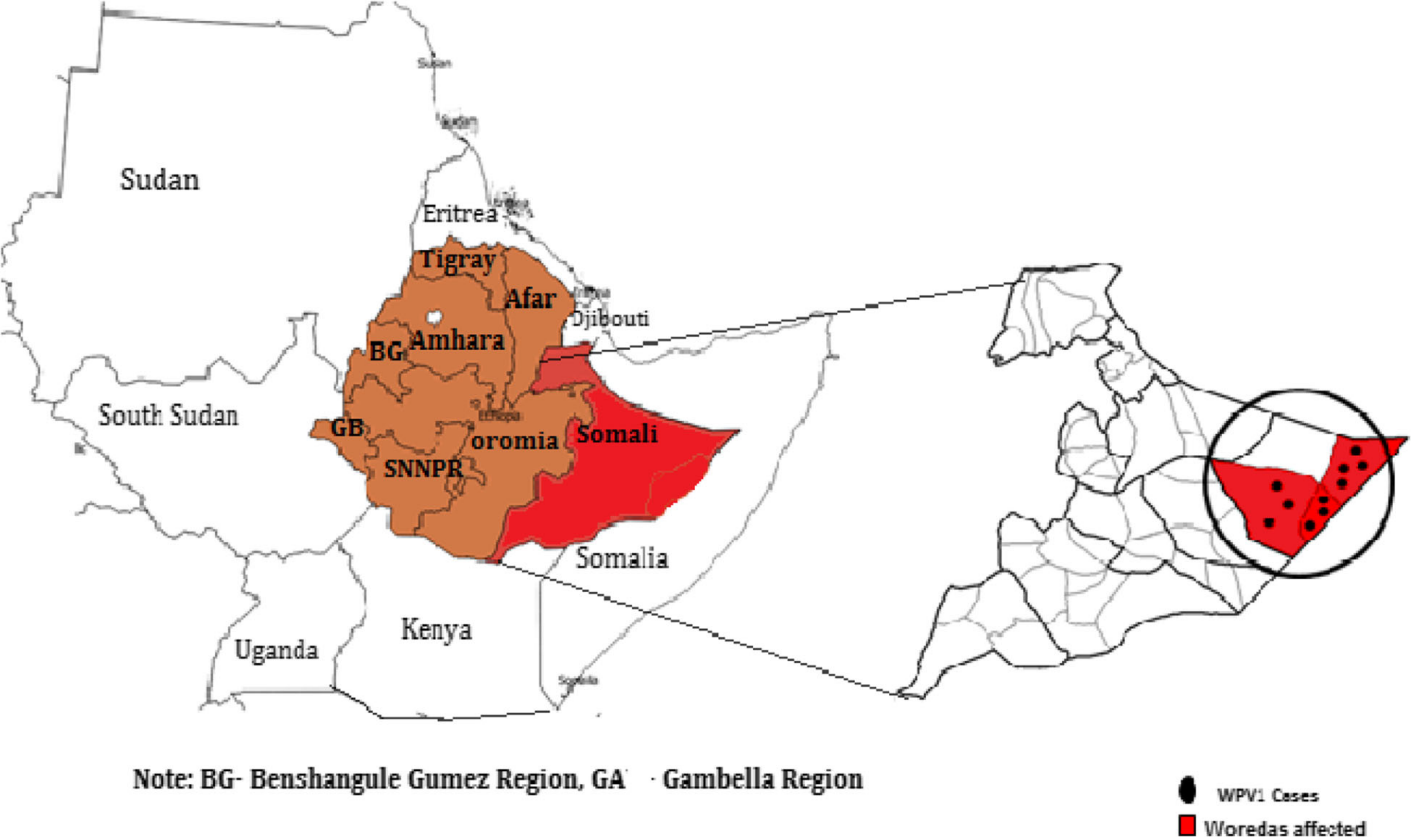
Wild polio virus affected Somali region and distribution of WPV1 cases in affected woredas of Dollo zone July 10, 2013- January 5, 2014

Somali region is located in the eastern and Southeastern part of Ethiopia. The region has common boundaries with Afar region and the Republic of Djibouti in the North, Kenya in the South, Oromia region in the West, and Somalia in the East and in the South. Administratively, the Somali Region is subdivided into nine administrative zones, which are subdivided into 68 Woredas and four town administrations, and further subdivided into Kebeles(equivalent to villages), which are the lowest administrative unit. Most of the people in the Somali region lead a pastoral nomadic lifestyle and frequently move from place to place. Additionally, there is free cross-border movement with neighboring countries for economic and social reasons. Dollo zone is one of the nine zones in the region with five Woredas bordering Somalia with a long porous border (Fig. 1).

### Study subjects

The study population of this investigation included all children paralyzed by WPV type 1 infection and healthy children less than 5 years old living in the village where the WPV cases were detected. Additionally, youth, parents, religious leaders, and influential people in the villages were targeted for focus group discussions (FGDs).

### Study design

Data was extracted from different investigation reports which include routine, SIA and surveillance data. Quantitative and qualitative methods were used during investigation.

### Investigation

After verbal consent, all confirmed WPV1 cases were investigated by a team of investigators from different backgrounds using standard national case investigation forms that include clinical, social and epidemiological variables. Additionally, the surveillance and routine immunization performance and cold chain status in all nearby health institutions were reviewed. We also assessed knowledge of health workers on surveillance and immunization including active case search for additional cases of Acute Flaccid Paralysis (AFP) both in health facilities and in the community. Immunization status was assessed among children in the communities where WPV cases were confirmed. Polio supplementary immunization data were reviewed and 10 FGDs were conducted to assess awareness and attitudes of the community members on poliomyelitis illness, immunization, and AFP surveillance. A FGD guide with pre-defined discussion points was used to conduct each focus group which consisted of 8 to 10 members representing various community groups. The AFP surveillance and routine immunization database available were used to supplement the field findings. Analysis of AFP data is conducted regularly by the program to monitor progress made, and the data is shared on a weekly basis with technical staff and partners.

### Data analysis

Data extracted from different investigation reports was entered into Microsoft Excel for analysis, and we also reviewed the investigation reports that were submitted by different investigation teams and abstracted data using a data capturing form. We summarized all FGDs that were conducted in each investigation.

### Definition of terms

*Confirmed wild polio case:* A suspected case with WPV isolated from a stool sample.

*Suspected AFP case:* Any child below 15 years of age with sudden onset of weakness or floppiness of one or more limbs or any person of any age in whom a clinician suspects polio.

*High-risk areas:* Zones that have international borders are hard to reach, or have poor surveillance and immunization performance.

## Results

Between July 10, 2013, and January 5, 2014, a total of 10 children (7 males and 3 females) were paralyzed by laboratory-confirmed WPV type-1 in the Somali region of Ethiopia. All cases occurred in Dollo zone affecting only three Woredas (Bokh, Galadi and Warder); each Woreda reported 3 cases, while Bokh woreda reported 4 cases (Fig. 1).

The index case was from Injure kebele in Galadi Woreda with date onset of paralysis of 10 July, 2013, and was not vaccinated, and the two close contacts with index case were positive for WPV1. Before the onset of paralysis, the index case had a contact history with a child, who came from Bursalah (a border town) in Somalia on 29 June, 2013. Following the onset of paralysis, the index case was taken to Galkacyo General Hospital of Somalia (180 km away from Injiro Kebele of Ethiopia) to seek medical care where the child was suspected as an AFP case, and immediately two stool specimens were collected and shipped to KEMRI. Nine (90%) of the confirmed cases occurred between July 10, 2013 through November 30, 2013, and the last case had a date onset on January 5, 2014.

**Fig. 2:**
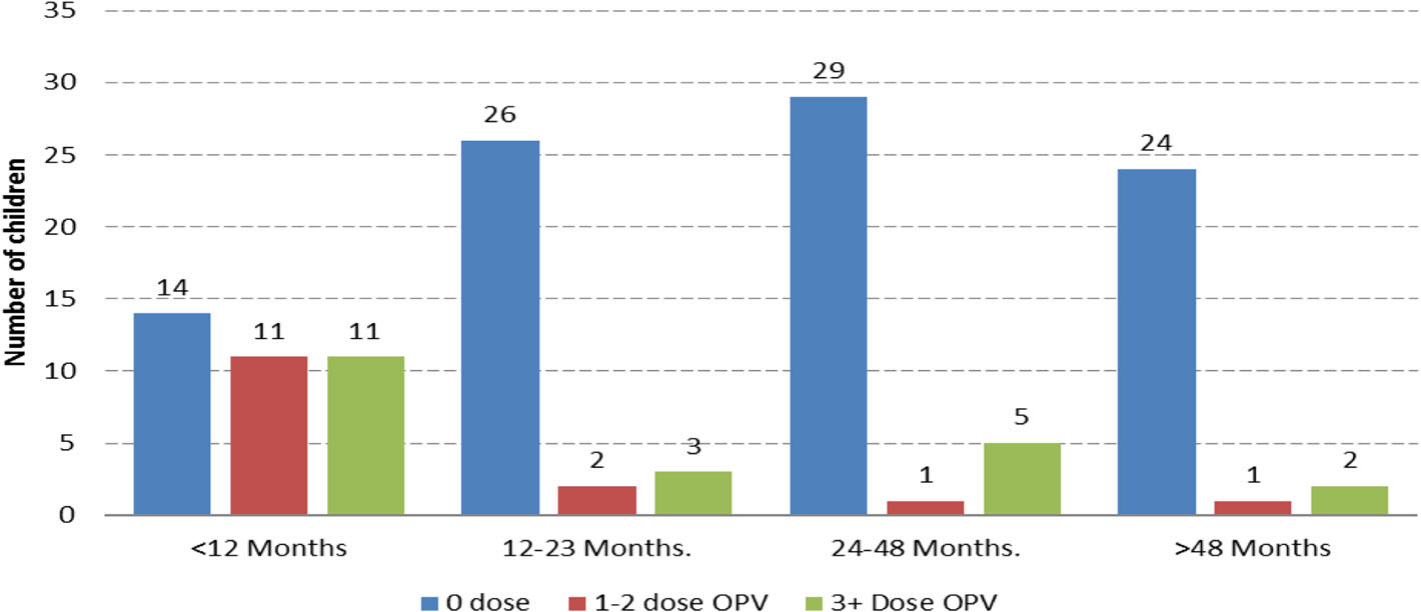
OPV vaccination status of sampled healthy children under 5 year’s old, Dollo zone, Somali Region, June 2013–January 2014

The average age of the children affected by polio was 31.5 months and ranged from three to 180 months; seven out of 10 cases were aged below 5 years old, while three were aged above 5 years old (8,11 and 15 years old). Seven of the affected children had never received any doses of OPV either in routine or during supplemental immunization before the onset of paralysis, while three had more than one dose of OPV. On the other hand, of the 129 healthy under 5 years old children assessed in the community, 93 (72%) had never received a dose of OPV (i.e. Zero doses of OPV), while 15 (12%) had received 1–2 doses of OPV. Over 80% of the children with zero doses were above 12 months old (Fig. 2). The routine immunization coverage in Dollo zone prior to the outbreak (as measured through OPV3 coverage) was 6% in 2012 and 24% in 2013, which increased 47% in 2014 and to 62 in 2015.

Timely investigation of the index case including the following cases (2nd, 3rd^,^ and 4th) within 72 h from notification was not attended, while the rest of the investigations met the standard time. Three of the cases (5th, 6th^,^ and 7th) investigated were children of nomads who actually lived in the bushes in small temporary huts and frequently traveled from place to place wherever there was grazing land; however, relatives of these children had frequent travel to Somalia for work and social reasons. Among the cases investigated, seven had a history of contact with someone or recent travel history to outbreak affected areas of Somalia. The clinical investigation indicates that almost all the cases (9) were associated with fever at onset of paralysis, and 8 of them had monoplegia, while the rest were hemiplegic.

**Fig. 3:**
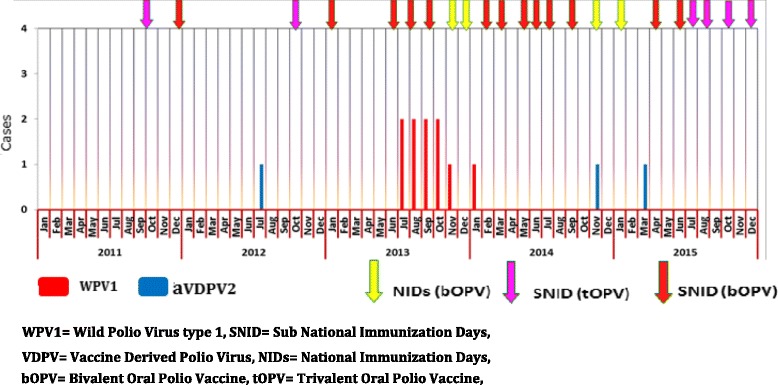
WPV1 onset and supplemental immunization response activities by month and year, January 2013–December 2015, Ethiopia

A total of 19 rounds of SIAs were conducted using trivalent and bivalent OPV including four full-scale national house-to-house campaigns using bivalent Oral Polio Vaccine (bOPV). Dollo, the outbreak affected zone, was covered in all the OPV rounds conducted between June, 2013 to December, 2015, however, only one round (using bOPV) had been conducted prior to the onset of paralysis of the index case, and Dollo was not covered during the preventive campaign conducted soon after the confirmation of the outbreak in Kenya (Fig. 3, Table 1). The SIAs in principle followed a one-month interval period, with delays in most instances, except in Nogob zone, where Short Interval Additional Doses (SIAD) were conducted with 1 week intervals (25/10/2014 AND 01/11/2014) to deliver two successive passages of bOPV to rapidly build population immunity. Nogob is one of the hard to reach and most inaccessible zone in Somali region including under developed infrastructure and poor health service access compared to other zones.

**Table 1:** Summary of immunization Responses before and after confirmation of the polio outbreak in Ethiopia June-Dec/2013

SN	Round	Areas covered	Target population	Age group	Administrative cov. (%0	Independent monitoring coverage(5)	Type of vaccine used
1	Immediate response-(June 5–8/2013)	Refugee campus and Dollo Ado host community	184,611	<15 years old	96	92	bOPV
2	Round 1(June 21–27/2013)	Somali region and 8 other high risk zones	2,664,894	<5 years old	95	91	bOPV(Somali)
							tOPV (others)
3	Round 2 (July 19–22/2013)	Somali region and 8 other high risk zones	2,664,894	<5 years old	96	NA	bOPV
4	Round 3 (Aug 30-Sep 12/2013)	Somali region except Nogob zone	846,934	<5 years old	95	NA	bOPV
5	Mop up activities (Aug to Oct/2013)	Chrati:	10,000	<15 years old	63	NA	bOPV
		Galadi:	12,000		99		
		Galadi and Bone:	20,000		109		
6	Border vaccination	28 locations on Somalia border	26513	<15 years old	NA	NA	tOPV
7	NID1 Oct. 3–6, 2013	Nation wide	1,288,175	<5 years old	99	93	tOPV
		Somali (16 Oct-10 Nov, 2013)	Doolo 108,459	<5 years old	90	NA	
			Nogob 65,843 (SIAD)		1stPassage-	89.3%	
			Other zones 784,975		2ndpassage (90%)	95%	
8	Nation wide Dec. 27–30, 2013	Nation wide	12,318,310(tOPV)	<5 years old	98.3%	NA	tOPV & bOPV
			3,152,961(bOPV)	<15 years old	92%		

Before the confirmation of the outbreak in Somali region and right after confirmed cases in Kenya, an immediate immunization response activity using bOPV was conducted (June 5–8, 2013), in the refugee camp of Dollo Ado and host community in Liben zone bordering Kenya. The 1st round (June 21–27, 2013) of SIA in the whole of Somali region including Dollo and 8 high risk zones in the country was conducted using bOPV, and the coverage was over 91% as measured by both Independent Monitoring(IM) and administrative coverage (Table 1).

**Fig. 4:**
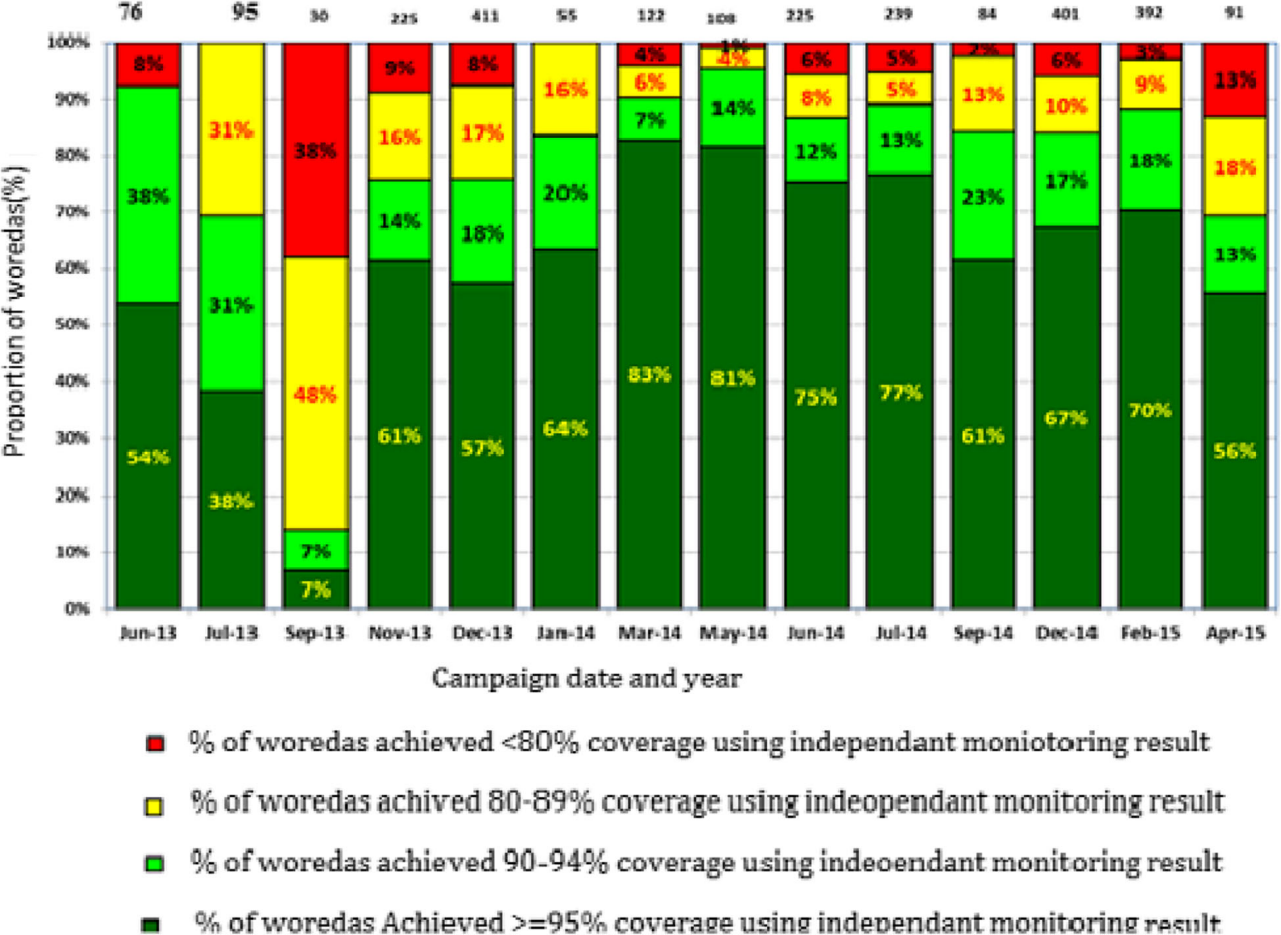
Woredas performance during Polio SIAs, 2013–2015, Ethiopia. Remarks: The number on top of the graph is the number of Woredas assessed during independent monitoring

The average administrative coverage of SIAs in Dollo zone was 91.4% (range 90% to 100%). The independent monitoring results indicate that the proportion of Woredas that achieved over 95% coverage ranged from 7 to 83%, and the average proportion of woredas that achieved 95% or above throughout the campaign period were only 60.8% (Fig. 4).

**Fig. 5:**
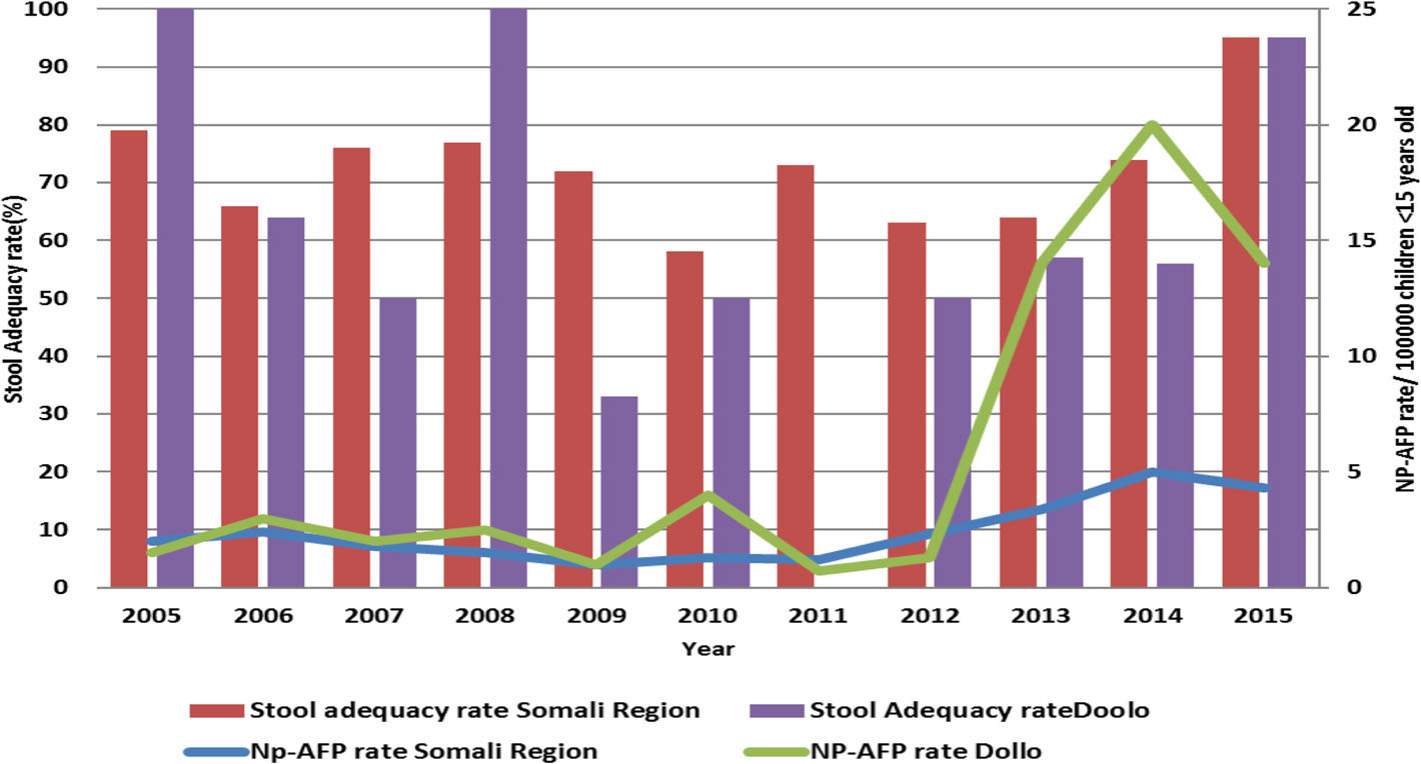
Trends of Non-polio AFP* and stool adequacy rate, Dollo zone and Somali Region, 2005–2015. *Non-polio AFP rate is calculated as number of non-polio AFP cases reported per 100,000 population <15 yrs

**Fig. 6:**
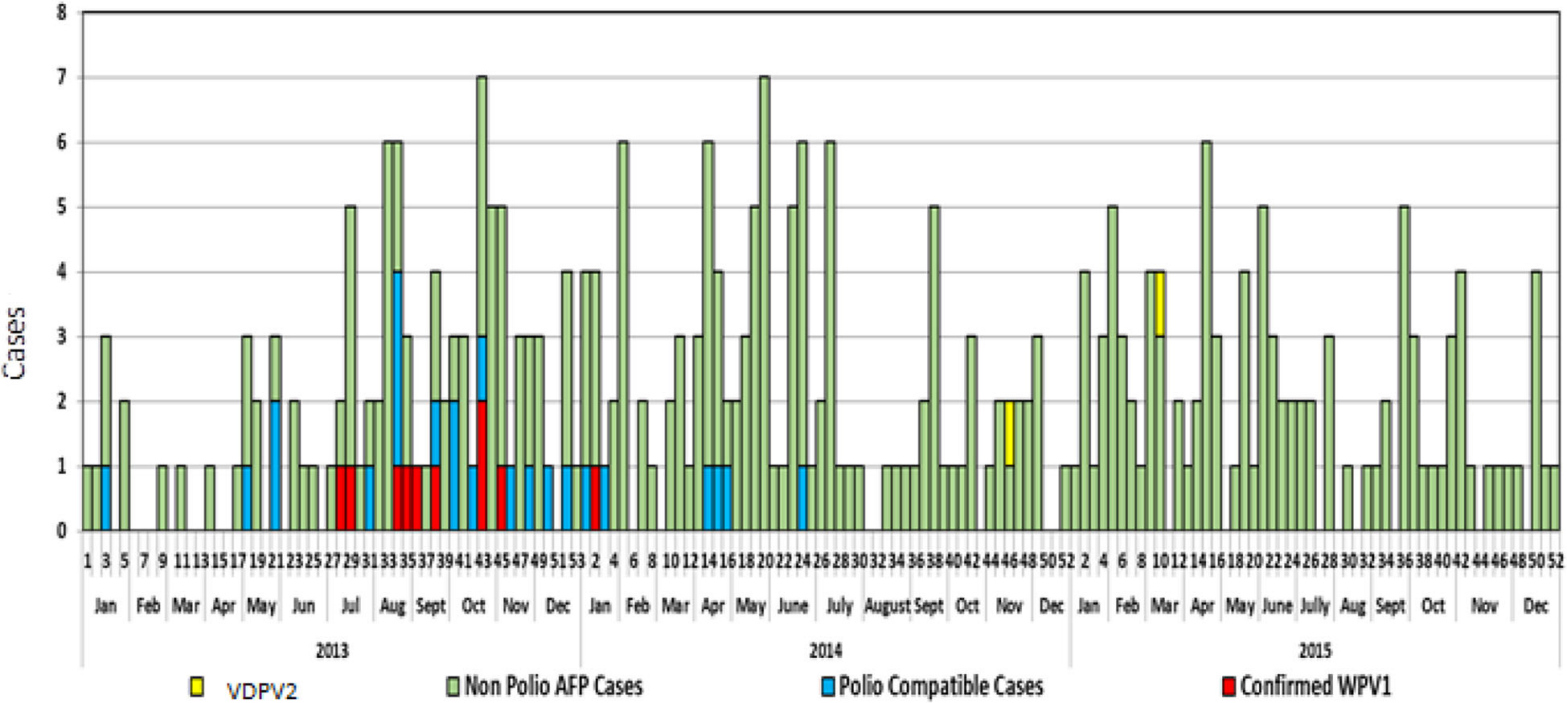
Classification of AFP cases in Somali region, Ethiopia, 2013–2015. aVDPV2: Ambiguous vaccine derived polio virus. Non polio AFP cases: these are cases discarded cases as the laboratory result is negative and they don’t look like polio during expert review. Polio compatible cases: A case that looks like polio on clinical ground and classified by expert committee. Confirmed case: Suspected cases of acute flaccid paralysis with laboratory confirmation of wild polio virus. AFP: Acute Flaccid Paralysis

Surveillance sensitivity in both Somali region and the outbreak affected Dollo zone was persistently low, and both had not achieved and maintained the two main surveillance performance indicators (Non-polio AFP rate of > 2 per 100,000 children below 15 years of age, and stool adequacy rate of ≥ 80% of AFP cases reported) in the years reviewed except in 2005 and 2008 for Dollo zone. It’s only since 2015 that both Dollo zone and Somali region achieved both indicators. In 2013 there were 18 AFP cases classified as polio-compatible in Somali region that four of them were prior to the outbreak started. In 2014 along with one WPV type 1 confirmation there were six AFP cases classified as clinical polio-compatible cases and one ambiguous Vaccine Derived Polio Virus type 2 (aVDPV2) case detected in Nogob zone and one aVDPV2 in 2015 in Dollo zones of Somali region. To rule out circulatory type of VDPV2 (cVDPV2), specimens were collected and tested among healthy children, but all were negative for wild polio virus or VDPV2 (Figs. 3, 5, 6).

All of the five woreda health offices assessed had neither specifically assigned surveillance nor immunization focal points, and there were no documented an independent surveillance activities conducted at woreda and health facility level. The routine immunization coverage in the zone was sub-optimal and health workers at the facility level were not trained on immunization prior to the outbreak started. Refrigerator temperature recording and monitoring for vaccines in all of the assessed health facilities was erratic, and only 50% of the facilities assessed had records of temperature monitoring over a 12 months period prior to the outbreak. However, no major cold chain failure or spoiled vaccines were found during investigation. The 10 FGDs conducted revealed that the demand for immunization is high and the participants of the FGD requested that routine immunization be given as a package rather than giving only polio vaccinations alone every round of the campaign.

## Discussion

After almost 5 years with no wild polio virus, Ethiopia again affected by polio outbreak importation in 2013. Low population immunity and population movement over the Ethiopia-Somalia border were the major predisposing factors for 2013 outbreak in Dollo zone to occur. The field investigation revealed that 7 out of 10 WPV cases hadn’t received any doses of OPV through routine or supplemental immunization activities, and we also found that 72% of healthy children below 5 years of age assessed from the communities had never received any doses of OPV. This suggests that the population immunity prior to the outbreak was already low and the mop up campaign conducted prior to the outbreak covered only Dollo Ado and the host community (Liben zone), and the outbreak affected Dollo zone was covered only with 1 round of SIAs using bOPV ahead of the outbreak confirmation in Dollo. This was insufficient to rapidly build population immunity and avert the outbreak as the sero-conversion rate of bOPV for a single dose is low on top of many inaccessible and hard to attain areas [15].

A 2012 coverage survey conducted before the outbreak indicated that the routine vaccination coverage in Somali region was below 31%, and in the presence of many high-risk groups, including nomadic pastoralists and mobile populations has no access to routine immunization services. This finding is consistent with other findings, which indicated that the routine immunization coverage in Somali Region had been below 50% for many years, including the first three to 6 months of the outbreak [16–18].

Most of the WPV cases had contacts from or travel history to outbreak affected districts of Somalia and within the affected woredas of Dollo zone, which may be connected with the importation to further transmission of WPV within the zone. Six of the 10 WPV cases were picked in Somalia by the Somalia surveillance team after crossing the international border for medical care, and this points to the lack of services in the border area indicating to suboptimal sensitivity of the surveillance system at the border and in hard to reach areas.

The outbreak occurred due to importation from the HOA (Somalia) and the genetic sequencing results indicated that the closest match was the virus circulating in Somalia, which was further closely related to the virus circulating in West Africa [18]. Prior to the 2013 outbreak, between 2004-2006 and 2008-2009 a similar importation of WPV occurred in the Somali region from Somalia following a “wild poliovirus importation belt” covering several countries extending from west Africa through central Africa to Horn of Africa countries [19, 20].

We found that surveillance sensitivity in Somali region as well in the affected zone (Dollo) had been sub-optimal for the last 10 years and was below the minimum expected AFP surveillance standard performance for the African region. The absence of surveillance and Expanded Program on Immunization (EPI) focal persons at the woreda health office level and the lack of independent active case search and sensitization activities by the focal persons may have contributed to the low sensitivity of the surveillance system until end of 2013. An external outbreak response assessment conducted 3 months following the outbreak confirmed the low sensitivity of the surveillance system in the affected zone [21]. The polio-compatible cases classified in 2012, 2013 and 2014 also support the findings of the gaps in the surveillance system in the region earlier and during the 3 months of the outbreak, and may suggest undetected circulation.

Seven out of the 10 cases were below 5 years old, which is similar to the findings of other studies, and affirms that polio affects children of younger age groups [3]. There was a delay in the outbreak investigation of the first (2nd, 3rd and 4th) cases beyond the standard 72 h of notification. The delay was because these AFP cases (that later turned out to be positive for WPV1) were initially detected and notified through the cross-border surveillance notification system after they had crossed to Galkacyo General Hospital of Somalia for medical care, where samples were collected and transported to polio laboratory in kenya(KEMRI). This created a delay in tracing the cases with gaps in available information. The presence of WPV in the healthy children of contacts also indicates the asymptomatic nature of the virus and affirms the already known phenomena that in every paralyzed child there might be around 200 asymptomatic children infected with wild polio virus.

Our study provides evidence that the SIAs, which were conducted in high-risk areas and in eight zones in the region following the confirmation of the outbreak in Somalia and Kenya may have been inadequate and/or of suboptimal quality and had gaps in accessing border areas and nomadic populations, despite the implementation of microplanning and independent monitoring of the quality of SIAs [10], and most importantly the history of previous outbreak route of transmission was not considered in preparation of preventive campaign. In Dollo zone, only one round of SIAs was conducted between the initial confirmation of the outbreak in Kenya and Somalia, and when the first case of WPV had date onset in Dollo zone. Considering the mobile nature of the population, many, inaccessible and hard to reach areas, population movement in and out, the immunity of the population was below the required to prevent the outbreak.

Although, the interest in immunization is high and the community requested for polio vaccination integrated with other antigens as a package rather than polio alone, the several hard to reach areas, nomadic lifestyle of the population and understaffed health service delivery system remain major obstacles to deliver vaccination services in many parts of the region. As Ethiopia is located in the so called polio importation belt, the country remains at risk of importations with Nigeria declared endemic once again in August 2016. This highlights the importance of maintaining high population immunity and sensitive surveillance to detect the low level of polio transmission.

We recognize one possible limitation of this study. Because all the investigations were conducted by a different team of investigators, and in different woredas at different times as cases were confirmed, this may affect the consistency of the investigations. However, to avoid bias, we used a standard semi-structured questionnaire and FGD guides to enable the investigating team to collect information in a standard way.

## Conclusion

We conclude that the importation of WPV in Somali region of Ethiopia may have been predisposed by a mix of risk factors including low population immunity fueled by chronic weaknesses in the delivery of routine immunization services throughout zone and in particular along the border with Somalia. Additionally, sub-optimal SIA quality and limited areas covered during SIAs may have contributed to the outbreak. Lack of a functional active surveillance system in the zone led to suboptimal surveillance sensitivity especially in the border areas. However, due to multiple SIAs campaigns of improved quality and enhanced surveillance, the outbreak was eventually successfully interrupted within 6 months of confirmation.

We recommend that the Somali Regional Health Bureau assign trained surveillance and immunization focal points at woreda health offices and health facilities, and conduct regular supportive supervision for surveillance and immunization with *an* emphasis on border areas and nomadic populations to prevent future importations. We further recommend instituting community-based surveillance using trained community volunteers in border areas and in the nomadic population to detect and notify cases in their area of residence. Apart from strengthening routine immunization service delivery and assigning trained immunization focal points, the Somali Regional Health Bureau is encouraged to establish the Reach Every Child (REC) strategy and conduct Periodic Intensification of Routine Immunization (PIRI) in hard to reach and mobile populations. We also recommend SIAs should reach every child, including children living in hard to reach areas, mobile and displaced populations using different innovative strategies like positioning permanent vaccination teams at water collection and cattle market points. Finally, surveillance and routine immunization, particularly in hard to reach areas, needs to be closely monitored in the region to be able to identify and understand the gaps in population immunity and surveillance for immediate remedial action to detect low level circulation and prevent future WPV outbreaks, especially given that there is still probable circulation of WPV in Nigeria.

## Abbreviations

AFP: Acute flaccid paralysis
FGDs: Focus group discussions
GPEI: The Global polio eradication initiative
KEMRI: Kenya Medical Research Institute
mOPV: Monovalent oral polio vaccine
NIDs: National Immunization Days
OPV: Oral Polio Vaccine
SIA: Supplemental Immunization Activities
SIAD: Short Interval Additional Doses
WHA: World Health Assembly
WPV: Wild polio virus
